# Target Localization in Wireless Sensor Networks Using Online Semi-Supervised Support Vector Regression

**DOI:** 10.3390/s150612539

**Published:** 2015-05-27

**Authors:** Jaehyun Yoo, H. Jin Kim

**Affiliations:** Department of Mechanical and Aerospace Engineering, Seoul National University, 599 Gwanangno, Gwanak-gu, Seoul KS013, Korea; E-Mail: yjh5455@snu.ac.kr

**Keywords:** semi-supervised learning, online support vector regression, wireless sensor network

## Abstract

Machine learning has been successfully used for target localization in wireless sensor networks (WSNs) due to its accurate and robust estimation against highly nonlinear and noisy sensor measurement. For efficient and adaptive learning, this paper introduces online semi-supervised support vector regression (OSS-SVR). The first advantage of the proposed algorithm is that, based on semi-supervised learning framework, it can reduce the requirement on the amount of the labeled training data, maintaining accurate estimation. Second, with an extension to online learning, the proposed OSS-SVR automatically tracks changes of the system to be learned, such as varied noise characteristics. We compare the proposed algorithm with semi-supervised manifold learning, an online Gaussian process and online semi-supervised colocalization. The algorithms are evaluated for estimating the unknown location of a mobile robot in a WSN. The experimental results show that the proposed algorithm is more accurate under the smaller amount of labeled training data and is robust to varying noise. Moreover, the suggested algorithm performs fast computation, maintaining the best localization performance in comparison with the other methods.

## 1. Introduction

Localization is one of the most important issues for wireless sensor networks (WSNs), because many applications need to know where the data have been obtained. For example, in a target tracking application, the measurement data are meaningless without location information about where the data were obtained.

With recent advances in wireless communications and electronics, localization using RSSI (received signal strength indicator) has attracted interest in many works in the literature [1,2]. Dealing with highly nonlinear and noisy RSSI, machine learning methods, such as neural network [3], Q-learning [4] and supervised learning [5], achieve good estimation for target localization. Because all of these methods require a large amount of the labeled training data for high accuracy, however, significant effort, such as cost, time and human skill, is needed. For example, in the indoor localization where GPS (Global Positioning System) is not available, the labeled training data points have to be collected by the human operator.

Against the need for a large set of labeled training data, semi-supervised learning has been recently developed. For example, in localization or tracking using WSN, an acquisition of labeled data may involve repeatedly placing a target and measuring corresponding RSSI from the sensor nodes in the known locations. On the other hand, the unlabeled data can be easily collected by recording RSSI without the position information.

A popular method to exploit unlabeled data is a manifold regularization (or graph-based method) that captures an intrinsic geometric structure of the training data and eventually makes the estimator smooth along the manifold [6]. The existing graph-based semi-supervised learning algorithms are mainly focused on classification problems (e.g., [7–13]), while only a few regression problems, such as localization, can be found in [14–16].

An advantage of the proposed method is its extension to online learning. Batch learning algorithms [16,17] cannot adjust to environmental changes, because they initially learn data only once. For example, in localization or tracking using WSN, the batch learning algorithms are not robust to changes in noise characteristics. To improve the robustness, these batch algorithms must be retrained from scratch, which has an expensive computational cost. To solve this problem, online learning methods, such as [18], are developed in order to update the learning model sequentially with the arrival of a new data point. They automatically track changes of the system model.

Motivated by the need for online learning, we extend the semi-supervised support vector regression (SVR) to the online learning framework that takes advantage of both semi-supervised learning and online learning. Online semi-supervised SVR (OSS-SVR) is accurate in that its solution is equivalent to the batch semi-supervised SVR. Contrary to other online algorithms [18,19], moreover, OSS-SVR can provide information on which data points are more important using support values [20]. These values are utilized when removing data in order to manage data storage, maintaining the good estimation performance.

We evaluate the developed algorithm (OSS-SVR) to estimate an unknown location of a mobile robot in a WSN, by comparing with two existing semi-supervised learning algorithms (*i.e.*, semi-supervised colocalization [14], semi-supervised manifold learning [15]) and one online learning algorithm using a Gaussian process [19]. In comparison with these state-of-the-art methods, we confirm that our algorithm estimates the location most accurately with fewer labeled data by efficiently exploiting unlabeled data. Furthermore, OSS-SVR converges most rapidly to the best estimated trajectory when the environment is changed. The computation of the proposed algorithm is very fast, maintaining the best accuracy.

This paper is organized as follows. In Section 2, we review some existing work for localization in a wireless sensor network. Section 3 overviews the technical structure for our localization problem. Section 4 presents the semi-supervised support vector machine algorithm. Section 5 describes an extension to the online version from the batch algorithm in Section 4. Section 6 reports empirical results with the description of the comparative algorithms, the parameter settings and the performance indices. Finally, concluding remarks are given in Section 7.

## 2. Related Work

Machine learning for localization aims to learn a model that defines the mapping function between sensor measurements and the location of a target of interest. However, due to the nonlinear and noisy characteristics of sensors, such as RSSI, sufficient labeled training data are needed for a good estimation. Furthermore, the algorithm should be able to update a learning model upon arrival of a new data point, in order to adjust to dynamic environments. We review some existing work based on whether they adopt the semi-supervised learning or online learning for the localization as follows.

Yang *et al.* [15] develop a semi-supervised manifold learning that solves the optimization problem based on a least squares loss function and graph Laplacian. Because it solves simplified linear optimization, it is fast, but inaccurate. Moreover, an online version of this algorithm has not been developed.

There is an online method based on a Gaussian process (GP) [19]. Since the online GP of [19] uses only current sensor measurements as training data, it is fast and naturally online. However, a strong assumption of known sensor locations is needed.

An online and semi-supervised learning method named colocalization by Pan *et al.* [14] estimates target location, as well as the locations of sensor nodes. They merge singular value decomposition, graph Laplacian and the loss function into one optimization problem. However, many tuning parameters that affect the optimal solution can be a burden. Furthermore, when the algorithm is applied to online learning, it updates the model by approximation using harmonic functions [13]. Because the approximated model is different from the solution using batch optimization, it can lose accuracy.

We introduce an SVR-based learning algorithm, which takes advantage of both online learning and semi-supervised learning methods. It does not need the assumption of known sensor locations and provides a unique solution and sparseness [20], contrary to an artificial neural network and reinforcement learning [21]. It performs accurate and efficient online learning based on incremental and decremental algorithms. The incremental algorithm learns the model given a new data point, providing the same solution to the batch optimization. The decremental algorithm safely removes worthless or less important data in order to limit the data size.

## 3. Overview

This section overviews the problem formulation, including the experimental setup, the data collection, the localization structure and the extended localization structure for a mobile target. In advance, we summarize the notations of functions and variables that are used in this paper:
*i* ∈ {1,…, *l*}: an index in a labeled training dataset when a target is located at *l* different locations*y*_X_*_i_* ∈ 

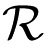
: the *i*-th target location in X coordinate*y*_Y_*_i_* ∈ 

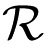
: the *i*-th target location in Y coordinate*z_ij_* ∈ 

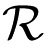
: an RSSI measurement of *j*-th sensor node corresponding to the *i*-th target location *y*_X_*_i_*, *y*_Y_*_i_**r_i_* = {*Z_i_*_1_,…, *Z_in_*} ∈ 

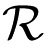
*^n^*: an RSSI observation set collected from all *n* sensor nodes
${\left\{r_{i},y_{\text{X}i}\right\}}_{i=1}^{l}$:a labeled dataset for X coordinate
${\left\{r_{i},y_{\text{Y}i}\right\}}_{i=1}^{l}$: a labeled dataset for Y coordinate
${\left\{r_{i}\right\}}_{j=1}^{u}$: an unlabeled dataset with the number of *u* data points*f*_X_ (*r* → *y*_X_) :the function mapping an RSSI measurement set *r* to X coordinate position*f*_Y_ (*r* → *y*_Y_) :the function mapping an RSSI measurement set *r* to Y coordinate position.

### 3.1. Experimental Setup

**Figure 1:**
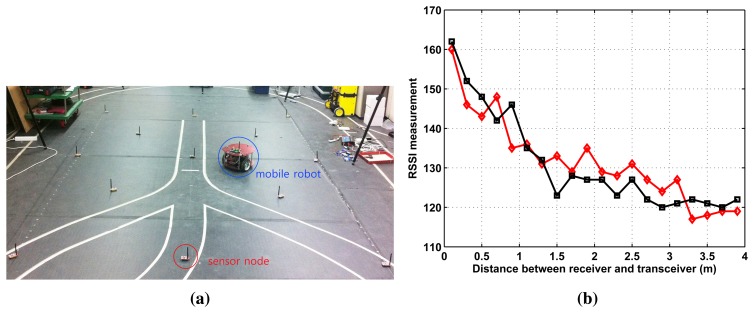
(**a**) Experimental setup with one mobile robot and thirteen sensor nodes. The nodes use a commercial CC2420 radio chip, which provides an IEEE 802.15.4 communication, and a received signal strength indicator (RSSI) for each received packet; (**b**) The relationship between RSSI measurement and distance between one fixed sensor node and one mobile node. The two trials are marked by a black-squared line and a red-diamond line, respectively.

Figure 1a shows the localization setup where 13 static sensor nodes are fixed in the 3 m × 4 m workspace. There is another base node (or base station) that is wirelessly connected to all deployed nodes. The role of the base station is to collect and record RSSI from the deployed nodes and to estimate a target position. A mobile robot equipped with one node broadcasts an RF signal whose strength (RSSI) is recorded by the sensor nodes. For accuracy analysis, the true location of the mobile robot is measured by the Vicon motion system that tracks reflective markers attached to the mobile robot.

The radio signal is easily influenced by the environment. Furthermore, its measured value affects the performance of the localization. Here, we examine the characteristics of RSSI measurement. In Figure 1b, we record RSSI measurements of one moving node (receiver), emitted by one fixed sensor node (transceiver). The distance between the transceiver and the receiver varies from 0.2 m to 4 m. The results of two trials show nonlinear and noisy RSSI measurements, which supports the need for learning to obtain accurate position estimates.

### 3.2. Data Acquisition

Semi-supervised learning uses both labeled and unlabeled datasets. For obtaining the labeled training data, *i.e.*, two sets ${\left\{r_{i},y_{\text{X}i}\right\}}_{i=1}^{l}$, ${\left\{r_{i},y_{\text{Y}i}\right\}}_{i=1}^{l}$, we collect RSSI measurements of the mobile robot placed at the different l locations, repeatedly. This collection process for the labeled dataset is called fingerprinting. On the other hand, the unlabeled dataset, *i.e.*, ${\left\{r_{j}\right\}}_{j=1}^{u}$, is obtained as we let the mobile robot move autonomously and collect only the RSSI measurements. Because the unlabeled dataset does not include the labels, *i.e.*, *y*_X_ and *y*_Y_, it is easy to collect a large amount of unlabeled data points. We note that the training dataset does not include the positions of the sensor nodes. Therefore, our algorithm does not need the positions of the sensor nodes.

### 3.3. Localization Structure

Our algorithm for the localization consists of three steps, *i.e.*, offline learning phase, test phase and online learning phase.

#### 3.3.1. Offline Learning Phase

Given the labeled and unlabeled training dataset, the major output of the training phase is two mapping functions *f*_X_ and *f*_Y_, which represent a relationship between RSSI observation set and the 2D position of a target. The details to obtain those functions will be described in Section 4.

#### 3.3.2. Test Phase

Test data r* ∈ 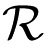
*^n^* are defined as an RSSI measurement set obtained from all *n* sensor nodes. Then, a base station estimates the location of the target using the test data r* by using the learned models *f*_X_ and *f*_Y_.

#### 3.3.3. Online Learning Phase

This paper considers a situation where new labeled data points are available after the offline learning phase. For example, landmark-based applications using RFID [22] or vision sensors [23] can obtain the online labeled dataset. The purpose of the online learning phase is to update the model *f*_X_ and *f*_Y_ when a new labeled data point comes. The detailed description is shown in Section 5.

### 3.4. Combination with Kalman Filter

In most target localization, targets move. However, the training datasets in the fingerprinting method do not include the velocity information of the target, because the training data are collected when the robot stops. One way to consider a moving target without the exact velocity measurement and without modifying the fingerprinting method is using a target's dynamic model in the framework of the Kalman filter. By defining the observation model as the estimated location obtained from the fingerprinting method, Kalman filter-based fingerprinting localization can be formulated. In Section 6.5, we compare the experimental results of the Kalman filter-based fingerprinting methods.

## 4. Semi-Supervised SVR

This section describes semi-supervised support vector regression. The models *f*_X_ and *f*_Y_ defined in the previous section are learned independently. For simplification, from this section, we omit the subscripts of *f*_X_, *f*_Y_ and *y*_X_, *y*_Y_.

### 4.1. Semi-Supervised Learning Framework

Given a set of l labeled samples ${\left\{\left(r_{i},y_{i}\right)\right\}}_{i=1}^{l}$ and a set of *u* unlabeled samples ${\left\{r_{i}\right\}}_{i=l+1}^{l+u}$, semi-supervised learning aims to establish a mapping *f* by the following regularized minimization functional:

(1) $$f^{*}=\arg\underset{f\in H_{k}}{\min}\frac{1}{l}\sum_{i=1}^{l}V\left(r_{i},y_{i},f\right)+\gamma_{A}{\left\|f\right\|}_{A}^{2}+\gamma_{I}{\left\|f\right\|}_{I}^{2}$$

where *V* is a loss function that will be defined in Equation (7), ${\left\|f\right\|}_{A}^{2}$ in Equation (3) is the norm of the function in the reproducing kernel Hilbert space (RKHS), ${\left\|f\right\|}_{I}^{2}$ in Equation (5) is the norm of the function in the low dimensional manifold and γ*_A_*, γ*_I_* are the regularization weight parameters.

By the representer theorem [24], the solution of Equation (1) can be defined as an expansion of kernel function over the labeled and the unlabeled data, given by:

(2) $$f\left(x\right)=\sum_{i=1}^{l+u}\alpha_{i}K\left(x_{i},x\right)+b$$

with the bias term *b* and the kernel function *K*(*x_i_*, *x_j_*) = 〈ϕ(*x_i_*), ϕ(*x_j_*)〉, where ϕ(·) is a nonlinear mapping to the RKHS.

The regularization term ${\left\|f\right\|}_{A}^{2}$, which is associated with the RKHS, is defined as:

(3) $${\left\|f\right\|}_{A}^{2}={\left(\Phi \alpha \right)}^{T}\left(\Phi \alpha \right)=\alpha^{T}K\alpha$$

where Φ = [ϕ(*x*_1_),…, ϕ(*x_l_*_+_*_u_*)], α = [α_1_,…, *α_l_*_+_*_u_*]*^T^* and *K* is the (*l* + *u*) × (*l* + *u*) kernel matrix whose element is *K_ij_*. We adopt the Gaussian kernel given by:

(4) $$K_{ij}=K\left(r_{i},r_{j}\right)=\exp\left(-{\left\|r_{i}-r_{j}\right\|}^{2}/\sigma_{k}^{2}\right)$$

where $\sigma_{k}^{2}$ is the kernel width parameter.

According to the manifold regularization, data points are samples from a low-dimensional manifold embedded in a high-dimensional space. This is represented by the graph Laplacian and the kernel function [6]:

(5) $$\begin{array}{ll} {\left\|\text{f}\right\|}_{I}^{2} & =\frac{1}{{\left(l+u\right)}^{2}}\overset{l+u}{\underset{i=1}{\text{∑}}}\overset{l+u}{\underset{j=1}{\text{∑}}}W_{ij}{\left(\text{f}\left(r_{i}\right)-f\left(r_{j}\right)\right)}^{2}\\ & =\frac{1}{{\left(l+u\right)}^{2}}f^{T}Lf \end{array}$$

where *L* is the graph Laplacian given by *L* = *D* − *W*, **f** = [*f*(*r*_1_),…, *f*(*r_l_*_+_*_u_*)]*^T^*, *W* is the adjacency matrix of the data graph and *D* is the diagonal matrix given by $D_{ii}=\sum_{j=1}^{l+u}W_{ij}$. In general, the edge weights *W_ij_* are defined as a Gaussian function of the Euclidean distance, given by:

(6) $$W_{ij}=\exp\left(-{\left\|r_{i}-r_{j}\right\|}^{2}/\sigma_{w}^{2}\right)$$

where $\sigma_{w}^{2}$ is the kernel width parameter.

Minimizing ${\left\|f\right\|}_{I}^{2}$ is equivalent to penalizing the rapid changes of the regression function evaluated between two data points. Therefore, $\gamma_{I}{\left\|f\right\|}_{I}^{2}$ in Equation (1) controls the smoothness of the data geometric structure.

We employ the following *ϵ*-insensitive loss function:

(7) $$V\left(r_{i},y_{i},f\right)=\{\begin{array}{ll} 0 & \text{if}\left|f\left(r_{i}\right)-y_{i}\right|<є\\ \left|f\left(r_{i}\right)-y_{i}\right|-є & \text{otherwise} \end{array}$$

Minimizing the loss function Equation (7) aims to bound the estimated error |*f*(*r_i_*) − *y_i_*| within the margin ϵ.

### 4.2. Complete Formulation

In this subsection, the complete formulation of the semi-supervised support vector regression is given based on the ingredients from Equations (2), (3), (5) and (7) to Equation (1). We additionally introduce slack variables ξ*_i_*, $\xi_{i}^{*}$ along with the є-insensitive loss function in order to cope with infeasible constraints of the optimization problem that uses only the є constraint. Substituting Equations (2) to (7) into Equation (1) with the slack variables ξ*_i_*, $\xi_{i}^{*}$, the primal problem is defined as:

(8) $$\begin{array}{r} \underset{\alpha \in R^{l+u},\xi \in R^{l},\xi *\in R^{l}}{\min}\frac{1}{l}\overset{l}{\underset{i=1}{\text{∑}}}\left(\xi_{i}+\xi_{i}^{*}\right)+\gamma_{A}\alpha^{T}K\alpha \\ +\frac{1}{{\left(l+u\right)}^{2}}\gamma_{I}\alpha^{T}\text{KLK}\alpha \\ \text{subject to}:y_{i}-\overset{l+u}{\underset{j=1}{\text{∑}}}\alpha_{j}K_{ij}-b\leq є+\xi_{i}\\ \overset{l+u}{\underset{j=1}{\text{∑}}}\alpha_{j}K_{ij}+b-y_{i}\leq є+\xi_{i}\\ \xi_{i},\xi_{i}^{*}\geq 0,i=1,\ldots ,l \end{array}$$

After the introduction of four sets of *l* multipliers β, β *, η and η*, the Lagrangian *H* associated with the problem is:

(9) $$\begin{array}{c} H(\alpha ,b,\xi ,\xi^{*},\beta ,\beta^{*},\eta ,\eta^{*})=\\ \frac{1}{2}\alpha^{T}\left(2\gamma_{A}K+\frac{2}{{\left(l+u\right)}^{2}}\gamma_{I}\text{KLK}\right)\alpha +\frac{1}{l}\overset{l}{\underset{i=1}{\text{∑}}}\left(\xi_{i}+\xi_{i}^{*}\right)\\ -\overset{l}{\underset{i=1}{\text{∑}}}\beta_{i}\left(є+\xi_{i}-y_{i}+\overset{l+u}{\underset{j=1}{\text{∑}}}\alpha_{j}K_{ij}+b\right)\\ -\overset{l}{\underset{i=1}{\text{∑}}}\beta_{i}^{*}\left(є+\xi_{i}^{*}+y_{i}-\overset{l+u}{\underset{j=1}{\text{∑}}}\alpha_{j}K_{ij}-b\right)\\ -\frac{1}{l}\overset{l}{\underset{i=1}{\text{∑}}}\left(\eta_{i}\xi_{i}+\eta_{i}^{*}\xi_{i}^{*}\right) \end{array}$$

In order to convert the primal problem to the dual representation, we take derivatives:

(10) $$\frac{\partial H}{\partial b}=\sum_{i=1}^{l}\left(\beta_{i}-\beta_{i}^{*}\right)=0$$

(11) $$\frac{\partial H}{\partial \xi_{i}^{\left(*\right)}}=\frac{1}{l}-\beta_{i}^{\left(*\right)}-\frac{1}{l}\eta_{i}^{\left(*\right)}=0$$

where $\beta_{i}^{\left(*\right)}$ denotes both β*_i_* and $\beta_{i}^{*}$.

Using Equations (10) and (11), we can rewrite the Lagrangian as a function of only α, β and β* from Equation (9), given by:

(12) $$\begin{array}{c} H\left(\alpha ,\beta ,\beta^{*}\right)=\\ \frac{1}{2}\alpha^{T}\left(2\gamma_{A}K+\frac{2}{{\left(l+u\right)}^{2}}\gamma_{I}\text{KLK}\right)\alpha -\alpha^{T}KJ_{L}^{T}B\\ -є\overset{l}{\underset{i=1}{\text{∑}}}\left(\beta_{i}+\beta_{i}^{*}\right)+\overset{l}{\underset{i=1}{\text{∑}}}y_{i}\left(\beta_{i}+\beta_{i}^{*}\right)\\ \text{subject to}:\overset{l}{\underset{i=1}{\text{∑}}}\left(\beta_{i}+\beta_{i}^{*}\right)=0\\ 0\leq \beta_{i},\beta_{i}^{*}\leq \frac{1}{l},i=1,\ldots ,l \end{array}$$

here, *J_L_* = [*I_l_*_×_*_l_* 0⃗*_l_*_×_*_u_*] is an *l* × (*l* + *u*) matrix, where *I_l_*_×_*_l_* is the *l* × *l* identity matrix and 0⃗*_l_*_×_*_u_* is the *l* × *u* zero matrix, and $B={\left[\beta_{1}-\beta_{1}^{*},\ldots ,\beta_{l}-\beta_{l}^{*}\right]}^{T}$.

Taking derivatives with respect to α, we obtain:

(13) $$\frac{\partial H}{\partial \alpha }=\left(2\gamma_{A}K+\frac{2}{{\left(l+u\right)}^{2}}\gamma_{I}\text{KLK}\right)\alpha -KJ_{L}^{T}B=0$$

From Equation (13), a direct relationship between α and 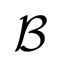 is obtained as follows:

(14) $$\alpha ={\left(2\gamma_{A}I+2\frac{1}{{\left(l+u\right)}^{2}}\gamma_{I}LK\right)}^{-1}J_{L}^{T}B$$

where *I* is the (*l* + *u*) × (*l* + *u*) identity matrix. Substituting Equation (14) back into the Lagrangian Equation (12), we arrive at the convex optimization problem:

(15) $$\begin{array}{c} \underset{\beta \in R^{l},\beta^{*}\in R^{l}}{\max}-\frac{1}{2}B^{T}J_{L}K{\left(2\gamma_{A}I+\frac{2}{{\left(l+u\right)}^{2}}\gamma_{I}LK\right)}^{-1}\\ J_{L}^{T}B-є\overset{l}{\underset{i=1}{\text{∑}}}\left(\beta_{i}+\beta_{i}^{*}\right)+\overset{l}{\underset{i=1}{\text{∑}}}y_{i}\left(\beta_{i}-\beta_{i}^{*}\right)\\ \text{subject to}:\overset{l}{\underset{i=1}{\text{∑}}}\left(\beta_{i}-\beta_{i}^{*}\right)=0\\ 0\leq \beta_{i},\beta_{i}^{*}\leq \frac{1}{l},\;i=1,\ldots ,l \end{array}$$

The optimal solution 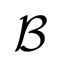
* of the above quadratic program is linked to the optimal α* in Equation (14). Then, the optimal regression function f*(*x*) can be obtained by substituting $\alpha^{*}={\left[\alpha_{1}^{*},\ldots ,\alpha_{l+u}^{*}\right]}^{T}$ into Equation (2). In summary, for the batch algorithm with given labeled and unlabeled datasets, the basic steps for obtaining the weights α* are: (I) construct edge weights *W_ij_* and the graph Laplacian *L* = *D* − *W*; (II) build a kernel function *K*; (III) choose regularization parameters γ*_A_* and γ*_I_*; and (IV) solve the quadratic programming Equation (15).

## 5. Online Semi-Supervised SVR

This section extends the semi-supervised batch SVR described in the previous section to online semi-supervised SVR. We use the idea from [25] for online extension, *i.e.*, adding or removing new data, maintaining the satisfaction of the Karush–Kuhn–Tucker (KKT) conditions. The resulting solution has the equivalent form to the batch version; thus, it does not learn the entire model again, which allows a much faster model update.

### 5.1. Karush-Kuhn-Tucker Conditions of Semi-Supervised SVR

We define a margin function *h*(*r_i_*) for the *i*-th data (*r_i_*, *y_i_*) as:

(16) $$h\left(r_{i}\right)=f\left(r_{i}\right)-y_{i}=\sum_{j=1}^{l}P_{ij}B_{j}-y_{i}+b$$

where *P* = *KQ* and $Q={\left(2\gamma_{A}I+\frac{2}{{\left(l+u\right)}^{2}}\gamma_{I}LK\right)}^{-1}$ Furthermore, the (*i*, *j*)-th element of the matrix *P* is defined as:

(17) $$P\left(r_{i},r_{j}\right)=P_{ij}=\sum_{k=1}^{l+u}K_{ik}Q_{kj}$$

where *K_ik_* = *K*(*r_i_*, *r_k_*) and *Q_kj_* = *Q*(*r_k_*, *r_j_*).

The Lagrange formulation of Equation (15) can be represented as:

(18) $$\begin{array}{ll} L_{D} & =\frac{1}{2}\overset{l}{\underset{i=1}{\text{∑}}}\overset{l}{\underset{j=1}{\text{∑}}}P_{ij}\left(\beta_{i}-\beta_{i}^{*}\right)\left(\beta_{j}-\beta_{j}^{*}\right)+є\overset{l}{\underset{i=1}{\text{∑}}}\left(\beta_{i}-\beta_{i}^{*}\right)\\ & -\overset{l}{\underset{i=1}{\text{∑}}}y_{i}\left(\beta_{i}-\beta_{i}^{*}\right)-\overset{l}{\underset{i=1}{\text{∑}}}\left(\delta_{i}\beta_{i}-\delta_{i}^{*}\beta_{i}^{*}\right)\\ & +\overset{l}{\underset{i=1}{\text{∑}}}\mu_{i}\left(\beta_{i}-\frac{1}{l}\right)+\overset{l}{\underset{i=1}{\text{∑}}}\mu_{i}^{*}\left(\beta_{i}^{*}-\frac{1}{l}\right)\\ & +b\overset{l}{\underset{i=1}{\text{∑}}}y_{i}\left(\beta_{i}-\beta_{i}^{*}\right) \end{array}$$

Computing partial derivatives of the Lagrangian *L_D_* leads to the KKT conditions:

(19) $$\frac{\partial L_{D}}{\partial \beta_{i}}=\sum_{j=1}^{l}P_{ij}\left(\beta_{j}-\beta_{j}^{*}\right)+є-y_{i}+b-\delta_{i}+\mu_{i}=0$$

(20) $$\frac{\partial L_{D}}{\partial \beta_{i}^{*}}=\sum_{j=1}^{l}P_{ij}\left(\beta_{j}-\beta_{j}^{*}\right)+є+y_{i}-b-\delta_{i}^{*}+\mu_{i}^{*}=0$$

(21) $$\frac{\partial L_{D}}{\partial b}=\sum_{j=1}^{l}\left(\beta_{j}-\beta_{j}^{*}\right)=0$$

(22) $$\begin{array}{cc} \delta_{i}^{\left(*\right)}\geq 0, & \delta_{i}^{\left(*\right)}\beta_{i}^{\left(*\right)} \end{array}=0$$

(23) $$\begin{array}{cc} \mu_{i}^{\left(*\right)}\geq 0, & \mu_{i}^{\left(*\right)}\left(\beta_{i}^{\left(*\right)}-\frac{1}{l}\right) \end{array}=0$$

(24) $$0\leq \beta_{i}^{\left(*\right)}\leq \frac{1}{l}$$

we recall $B_{i}=\beta_{i}-\beta_{i}^{*}$ in Equation (12) and define a margin function *h*(*x_i_*) for the *i*-th data (*x_i_*, *y_i_*) as:

(25) $$h\left(r_{i}\right)=f\left(r_{i}\right)-y_{i}=\sum_{j=1}^{l}P_{ij}B_{j}-y_{i}+b$$

By combining all of the KKT conditions from Equations (19) to (24), we can obtain the following:

(26) $$\{\begin{array}{ll} h\left(r_{i}\right)>є, & B_{i}=-\frac{1}{l}\\ h\left(r_{i}\right)=є, & -\frac{1}{l}<B_{i}<0\\ -є<h\left(x_{i}\right)<є, & B_{i}<0\\ h\left(r_{i}\right)=-є, & 0<B_{i}<\frac{1}{l}\\ h\left(r_{i}\right)<-є, & B_{i}=\frac{1}{l} \end{array}$$

According to Karush-Kuhn-Tucker (KKT) conditions, we can separate labeled training samples into three subsets:

(27) $$\begin{array}{ll} \text{Support set} & S=\left\{\left(r_{i},y_{i}\right)|0<\left|B_{i}\right|<\frac{1}{l},\left|h\left(r_{i}\right)\right|=є\right\}\\ \text{Error set} & E=\left\{\left(r_{i},y_{i}\right)|\left|B_{i}\right|<\frac{1}{l},\left|h\left(r_{i}\right)\right|>є\right\}\\ \text{Remaining set} & R=\left\{\left(r_{i},y_{i}\right)|\left|B_{i}\right|<0,\left|h\left(r_{i}\right)\right|<є\right\} \end{array}$$

### 5.2. Adding a New Sample

Let us denote a new sample and corresponding coefficient by (*r_c_*, *y_c_*), 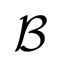
*_c_* whose initial value is set to zero. When the new sample is added, 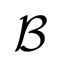
*_i_* (for *i* = 1,…,*l*), *b* and 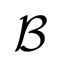
*_c_* are updated. The variation of the margin is given by:

(28) $$\Delta h\left(r_{i}\right)=\sum_{j=1}^{l}P_{ij}\Delta B_{j}+P_{ic}\Delta B_{c}+\Delta_{b}$$

The sum of all of the coefficients should remain zero according to Equation (10), which, in turn, can be written as:

(29) $$\Delta B_{c}+\sum_{i=1}^{l}\Delta B_{i}=0$$

By the KKT conditions in Equation (27), only the support set samples can change 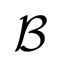
*_i_*. Furthermore, for the support set samples, the margin function is always є, so the variation of the margin function is zero. Thus, Equations (28) and (29) can be represented to discover the values of the variations of 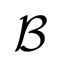 and *b*, in the following:

(30) $${\left[\begin{array}{cccc} \Delta b & \Delta B_{s_{1}} & \cdots & \Delta B_{s_{l_{s}}} \end{array}\right]}^{T}=\kappa \Delta B_{c}$$

where:

(31) $$\kappa ={\left[\begin{array}{cccc} \kappa_{b} & \kappa_{s_{1}} & \cdots & \kappa_{s_{l_{s}}} \end{array}\right]}^{T}$$

(32) $$=-M{\left[\begin{array}{cccc} 1 & P_{s_{1}c} & \cdots & P_{s_{l_{s}}c} \end{array}\right]}^{T}$$

and:

(33) $$M={\left[\begin{array}{cccc} 0 & 1 & \cdots & 1\\ 1 & P_{s_{1}s_{1}} & \cdots & P_{s_{1}s_{l_{s}}}\\ \vdots & \vdots & \ddots & \vdots \\ 1 & P_{s_{l_{s}}s_{1}} & \cdots & P_{s_{l_{s}}s_{l_{s}}} \end{array}\right]}^{-1}$$

This states that we can update 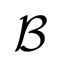
*_i_* for (*r_i_*, *y_i_*) ∈ *S* and *b* when 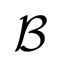
*_c_* is given. Moreover, *h*(*r_i_*) for (*r_i_*, *y_i_*) ∈ *S* are consistent according to Equation (27).

### 5.3. Recursive Update of Inverse Matrix

Calculating the inverse matrix Equation (33) can be inefficient. Fortunately, Ma *et al.* [25] and Martin [26] suggest a recursive algorithm to update *M* Equation (33) without explicitly computing the inverse. This method also can be used for our algorithm. When a sample (*r_i_*, *y_i_*) is added to the set *S*, the new matrix *M* can be updated in the following:

(34) $$M^{\text{new}}=\left[\begin{array}{cc} M & \vec{0}\\ {\vec{0}}^{T} & 0 \end{array}\right]+\frac{1}{\nu_{i}}\left[\begin{array}{c} \kappa_{M}\\ 1 \end{array}\right]\;\left[\begin{array}{cc} \kappa_{M}^{T} & 1 \end{array}\right]$$

where:

(35) $$\kappa_{M}=-M{\left[\begin{array}{cccc} 1 & P_{is_{1}} & \cdots & P_{is_{l_{s}}} \end{array}\right]}^{T}$$

(36) $$\nu_{i}=P_{ii}+\left[\begin{array}{cccc} 1 & P_{is_{1}} & \cdots & P_{is_{l_{s}}} \end{array}\right]\kappa_{M}$$

and 0⃗ is the (*l_s_* + 1) × 1 zero vector.

When the *k*-th sample (*r_k_*, *y_k_*) in the set *S* is removed, *M* can be updated by:

(37) $$M_{ij}^{\text{new}}=M_{ij}-M_{ik}M_{kj}/M_{kk}$$

for *i*, *j* ∈ [1, ⋯, *k*, *k* + 2, ⋯, *l_s_* + 1].

### 5.4. Incremental and Decremental Algorithms

Since the standard online SVR was developed in [25], many other researches, such as [27–29], had followed its incremental and decremental algorithms that update the variation Δ 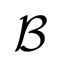
*_c_* caused by a new sample or a removed sample, for the quick termination of the online learning. The only difference in comparison with the existing online SVR is making the kernel matrix *K* and the graph Laplacian *L* before starting the incremental and decremental algorithms. Therefore, the same incremental and decremental algorithms can be used once we build *K* and *L*. We skip the details (see Section 3.2 and Appendix in [25]) to avoid overlap.

We close this section with remarks on the usages of the incremental and decremental algorithms. The incremental algorithm is used whenever a new data point comes in. However, maintaining all data whose volume increases over time is inefficient for calculation speed and memory. As a solution to this problem, the decremental algorithm is used to limit the data size by forgetting useless or less important data. The concept of support vectors offers a natural criterion for this. The useless dataset is defined as the training data in the set *R*. Furthermore, less important data are defined as the training data within *S* ∪ *E*, which have small coefficients 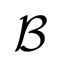
*_i_* that slightly affect the margin function in Equation (25). The relationship between localization performance and calculation time, when adopting the decremental algorithm, will be analyzed in Section 6.3.

## 6. Experiments

For evaluation of the online learning algorithms, we assume that the target location corresponding RSSI measurements at any time are available as labeled training data.

This section describes the comparative algorithms in Section 6.1 and the parameter setting in Section 6.2. In Section 6.3, localization results are shown when we vary the initial training data amount and intentionally change an environment. In Section 6.4, the results are shown when we apply decremental algorithm. In Section 6.5, Kalman filter-based fingerprinting methods are compared.

### 6.1. Comparative Algorithms

The proposed algorithm is compared with other recently-developed learning algorithms, summarized in Table 1.

#### 6.1.1. Semi-Supervised Colocalization

Similarly to our algorithm, this builds an optimization problem with a loss function and graph Laplacian. As a training set, semi-supervised colocalization (SSC) uses target location, locations of sensor nodes and RSSI measurements. Given RSSI measurements as test data, SSC estimates the location of the target and also the locations of the sensor nodes in order to recover unknown locations of the sensor nodes in the training set. For better target tracking performance, many labeled (known) locations of sensor nodes are needed.

#### 6.1.2. Gaussian Process

For RSSI-based localization, a Gaussian process makes a probabilistic distribution that represents where the target is located. Similar to SSC, the online GP of [19] also needs an assumption that the locations of all sensor nodes are known, while the SVR-based method does not need to know the sensor locations. We give SSC and GP advantageous information of the locations of the sensor nodes for all experiments. Localization using GP in a wireless sensor network is studied in our previous work.

#### 6.1.3. Semi-Supervised Manifold Learning

Semi-supervised manifold learning (SSML) is extended from Laplacian regularized least squares (LapRLS [30]) by modifying a classification problem to a regression problem. Because this method uses fast linear optimization, it can be suitable for real-time applications. However, this is inaccurate, and only the batch version has been reported. In order to compare online tracking performance dealing with new incoming data, we perform SSML whenever a new data point comes, enduring a long calculation time.

### 6.2. Parameter Setting

**Figure 2:**
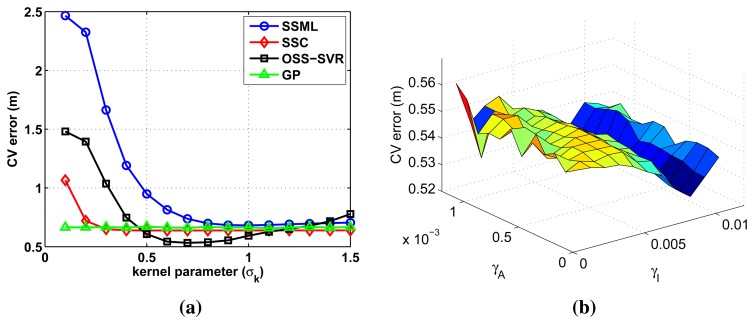
CV (cross-validation) error over the validation set as (**a**) a function of the kernel parameter for all compared methods and (**b**) a function of regularization parameters γ*_I_*, γ*_A_* for online semi-supervised (OSS)-SVR.

All tuning parameters are optimized by 10-fold cross-validation [31] using 40 labeled data and 30 unlabeled data. We set optimal kernel parameters minimizing the training error. Figure 2 shows the training error curves of the used algorithms with respect to the tuning parameters. The training error is defined as CV (cross-validation) error, given by:

$$\begin{array}{c} \text{CV error}=\frac{1}{K}\overset{K}{\underset{k=1}{\text{∑}}}E_{k}\left(\lambda_{1},\lambda_{2},\ldots \right)\\ E_{k}\left(\lambda_{1},\lambda_{2},\ldots \right)=\sqrt{\frac{1}{N}\overset{N}{\underset{i=1}{\text{∑}}}{\left\|{\hat{y}}_{i}-y_{i}\right\|}_{2}^{2}} \end{array}$$

where *K* = 10 is the number of the fold, *E_k_* is the RMSE (root mean squared error) of one fold, *N* is the number of data in one fold, *ŷ_i_* is the estimated location, *y_i_* is the true location and ‖·‖_2_ denotes the vector two-norm. Variables of CV error are defined as λ_1_ = σ*_k_* in Figure 2a and λ_1_ = γ*_I_*, λ_2_ = γ*_A_* in Figure 2b.

All of the compared learning algorithms use common kernel function Equation (4). The kernel parameter σ*_k_* in Equation (4) is selected to be smaller than 1.5, avoiding extremely small values, which yield large RMSE in all of the methods, as shown in Figure 2a. The parameter є in Equation (7) is set to 0.02, after the similar analysis in the range [0.01, 1]. Small changes in the tuning parameters do not affect training error much.

The parameters γ*_I_* and γ*_A_* are additional parameters for OSS-SVR, as described in Section 4. As γ*_I_* increases, the influence of unlabeled data increases by Equation (1). Therefore, γ*_I_* determines the impact of unlabeled data. As can be seen in Figure 2b, γ*_I_* is more sensitive than γ*_A_*. Therefore, we find a proper range of γ*_A_* first and, then, select good values for γ*_I_*, γ*_A_*.

In general, the graph Laplacian Equation (6) is built with *k*-nearest neighbor data points. When the graph Laplacian with *k*-nearest neighbors is updated online with new data, however, calculation of the new graph Laplacian is quite slow, because it has to find new neighbors and make new kernel matrices. This has to be repeated while Δ 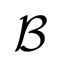
*_i_*, Δ*b*, Δ 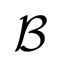
*_c_* and Δ*h*(*x_i_*) are being updated using Equation (30). To avoid this complex process, we build the graph Laplacian with all data points instead of the *k*-nearest data points. Therefore, we can quickly update the graph Laplacian with the additional advantage of no need for finding optimal parameter *k*.

### 6.3. Localization Results for a Circular Trajectory

In this section, we test localization algorithms where the target moves along a circular path. First, we show the online tracking results of each algorithm with respect to the repeated target motion. Second, we compare performance varying the amount of initial training data. Third, we examine localization performance when the system model is disturbed by bias noise.

**Figure 3:**
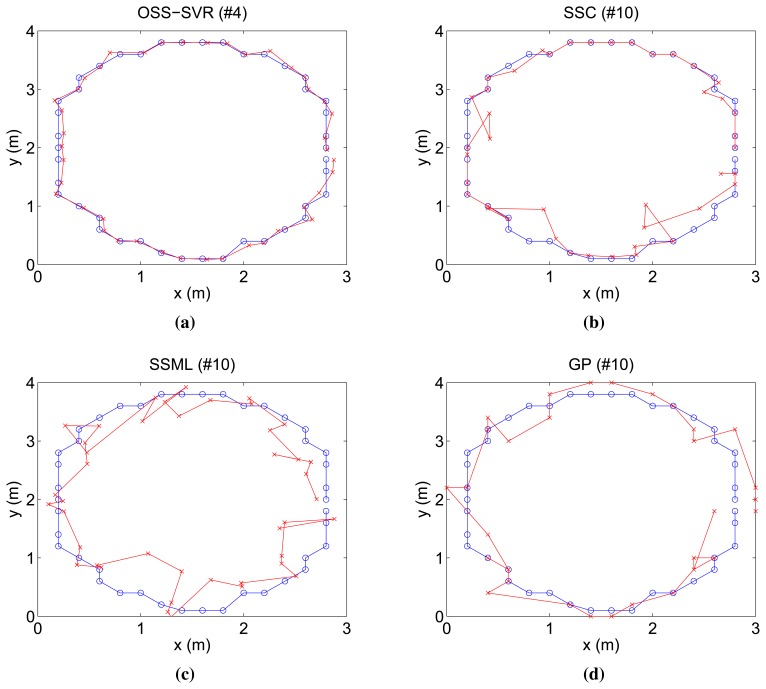
Localization results of a circular trajectory for four different methods. Target circles ground repeatedly, whose true trajectory is marked by the blue-circled line, and the estimated path is shown in red line. All of the figures show online tracking results where the title number of each figure indicates the number of laps the target has made.

First, Figure 3 shows the online tracking result when no initial labeled data and 30 initial unlabeled data points are used for each algorithm. Moreover, a new labeled data point is learned per time step. The number on the title of each figure indicates how many laps the target has moved. Overall, localization performances, except GP, tend to improve over time, because they learn the repeated relationship between RSSI measurements and target positions. In this experiment, we compare the capability of the algorithms for accuracy and speed of convergence to the true trajectory. In Figure 3, although 10 repetitions passed, SSC, SSML and GP do not approach the true trajectory. On the other hand, our algorithm gets close to the true trajectory in only four repetitions. In comparison with all of the others, the proposed online semi-supervised SVR (OSS-SVR) gives the best accurate localization with the fastest convergence.

**Figure 4:**
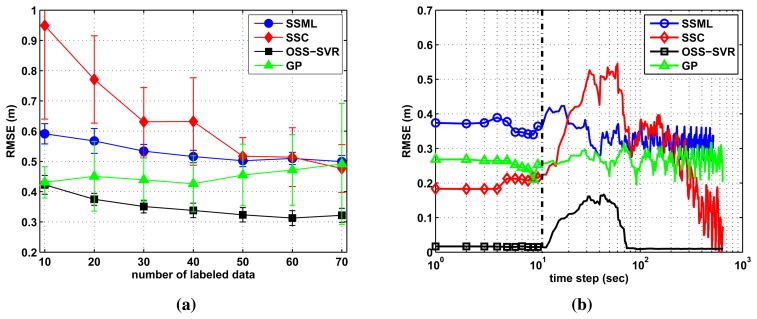
Localization performance of circular trajectory according to (**a**) the number of labeled data and (**b**) the change of the model interrupted by bias noise.

Next, we vary the initial number of labeled training data and show localization error during two laps of the target motion. Localization error is defined as the root mean squared error (RMSE), given by:

(38) $$\text{RMSE}=\sqrt{1/T\sum_{t=1}^{T}{\left\|{\hat{y}}_{t}-y_{t}\right\|}_{2}^{2}}$$

where ‖·‖_2_ denotes the vector two-norm, *ŷ_t_* is the estimated location, *y_t_* is the true location at time step *t* and *T* is the duration of two laps of the target moving. For all of the methods, the same unlabeled data (total 30 points) are used along with the randomly-picked labeled data. Figure 4a shows the mean-standard deviation over 10 repetitions in order to reduce the statistical variability of the randomly-picked labeled data. We can observe that our method gives the smallest error and deviation over the variation of labeled data.

Finally, when the environment is changed, we examine the robustness or fast recovery to an accurate solution of each online learning. Figure 4b shows how fast and accurate they learn the changed model under the same experimental setup used in Figure 3. At this time, localization error is defined by $\text{RMSE}=\sqrt{1/20\sum_{t=i-19}^{i}{\left\|{\hat{y}}_{t}-y_{t}\right\|}_{2}^{2}}$, with current time step *i*. We intentionally force constant bias noise, whose magnitude is 10% of the maximum RSSI value, into the measurements of one sensor node. The bias noise is added (*i.e.*, at 10 seconds in Figure 4b) just after the learning models of each algorithm have converged. Our method quickly builds a corrected learning model leading to the best estimation, while SSC shows large error to the bias noise and slow recovery. We note that SSML and GP are not much affected by bias noise, because the online GP of [19] uses only current sensor measurement and SSML trains all of the old and current data per every time step. Therefore, the bias noise does not have meaning for the two methods. However in the long run, they cannot approach accurate estimation in comparison with SSC and OSS-SVR.

### 6.4. Localization Results for a Sinusoidal Trajectory

**Figure 5:**
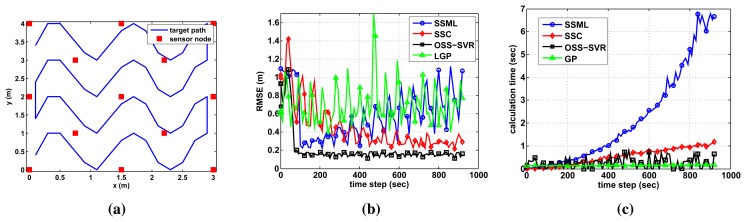
Localization performance of a sinusoidal trajectory, when we apply a decremental algorithm to each method in order to limit the amount of training data. (**a**) Target path; (**b**) localization error; and (**c**) calculation time.

In the experimental setting of the prior section, the target moved in a circular trajectory in order to show visual localization results. In this section, a more complex target path is made, as in Figure 5a, to search the whole area. In the case of the online learning, many training data points stack up. Therefore, we limit the amount of training data using a decremental algorithm. For OSS-SVR, a data point having the lowest support value is removed. For the other methods, the oldest data point is removed sequentially. Those data points are removed when the RMSE is smaller than a pre-defined threshold value. We set 0.3 for OSS-SVR and SSC and 1.0 for SSML as the threshold values, because each method has different values for the final error. Figure 5 shows the online tracking results when no initial labeled data and 30 initial unlabeled data points are used. As shown in Figure 5b, OSS-SVR yields the best accuracy with the fastest convergence. During the early time steps, OSS-SVR may take a slightly longer time than the others in Figure 5c. As time passes, however, the computation time remains bounded to being small, because the well-learned model does not spend much time for learning the new training data.

### 6.5. Localization Results of Kalman Filter-Based Fingerprinting

Fingerprinting methods can be further improved for tracking a moving target. This section shows an extended localization algorithm where the fingerprinting methods are combined with the Kalman filter. The target state takes the form *T_t_* = [*y*_X_*_t_*, *y*_Y_*_t_*, *v*_X_*_t_*, *v*_Y_*_t_*]*T* where *y*_X_*_t_*, *y*_Y_*_t_* are 2D positions and *v*_X_*_t_*, *v*_Y_*_t_* are 2D velocities. The state dynamics are given by:

(39) $$T_{t}=FT_{t-1}+\Gamma_{\sigma }$$

where four-by-four matrix *F* is given by [1 0 Δ*t* 0; 0 1 0 Δ*t*; 0 0 1 0; 0 0 0 1] and Γ_σ_ is a Gaussian noise, whose mean is zero and variance is Σ_σ_. The variance matrix Σ_σ_ is a diagonal matrix whose diagonal elements are $\sigma_{\text{X}}^{2}$, $\sigma_{\text{Y}}^{2}$, $\sigma_{v_{\text{X}}}^{2}$ and $\sigma_{v_{\text{Y}}}^{2}$, respectively.

We use the estimated position obtained from the fingerprinting methods as the observation of the Kalman filter, *i.e.*, *Z_t_* = [*ŷ*_X_*_t_*, *ŷ*_Y_*_t_*]*^T^*, where *ŷ*_X_*_t_*, *ŷ*_Y_*_t_* are the estimated position from the fingerprinting learning method, such as OSS-SVR, SSC, SSML and GP. Therefore, the measurements are given by:

(40) $$Z_{t}=HT_{t}+\Gamma_{є}$$

where two-by-four matrix *H* is given by [1 0 0 0; 0 1 0 0] and Γ_є_ is a Gaussian noise, whose mean is zero and variance is Σ_є_. The variance matrix Σ_є_ is a diagonal matrix whose diagonal elements are є^2^. Then, iterative updates of dynamic and observation model are implemented.

**Figure 6:**
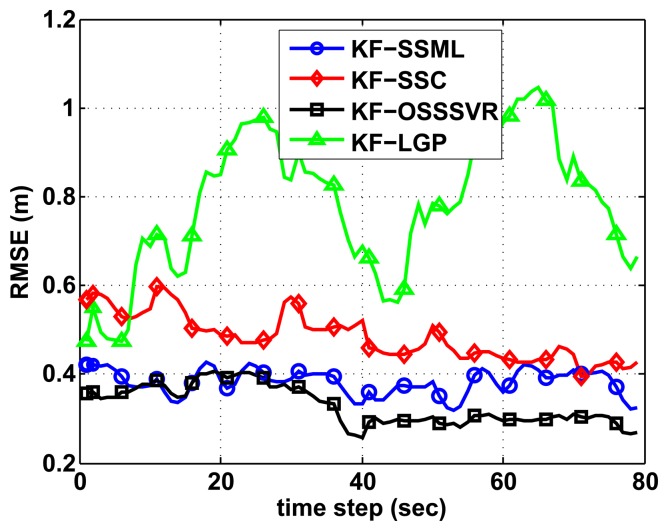
Comparison of Kalman filter-based localization of SSML, GP and our algorithm.

We compare the results of the Kalman filter-based localization of SSML, GP, SSC and our algorithm. The experimental trajectory is same as Figure 5a. In this scenario, we use 100 initial labeled training data points and 50 initial unlabeled data points. As shown in Figure 6, all of the Kalman filter-based localization results provide better accuracy than the basic fingerprinting methods in Figure 5b. Furthermore, in Figure 6, the suggested algorithm gives the greatest accuracy when the Kalman filter is combined.

## 7. Conclusions

This paper proposes an online semi-supervised regression algorithm by combining the core concepts of the manifold regularization framework and the supervised online SVR. By taking advantage of both semi-supervised learning and online learning, our algorithm achieves good accuracy using only a small number of labeled training data and automatically tracks the change of the system to be learned. Furthermore, support vectors are used to decide the importance of a data point in a straightforward manner, allowing minimal memory usage. In comparison with the three state-of-the-art learning methods for target localization using WSN, the proposed algorithm yields the most precise performance of online estimation and rapid recovery to accurate estimation after bias noise is added. Moreover, computation of the suggested algorithm is fast, while maintaining the best accuracy in comparison with the other methods. Furthermore, we formulate a Kalman filter-based fingerprinting localization in order to track a moving target more smoothly and accurately.

## Figures and Tables

**Table 1:** Comparative algorithms. SVR, support vector regression.

**Learning Method**	**Semi-Supervised**	**Online**
Semi-Supervised Manifold Learning [15]	○	×
Gaussian Process [19]	×	○
Semi-Supervised Colocalization [14]	○	○
Online Semi-Supervised SVR (developed)	○	○
